# Supplementary figures and images for: Multiple resistance factors collectively promote inoculum-dependent dynamic survival during antimicrobial peptide exposure in Enterobacter cloacae
Source: PLoS Pathog. 2024 Aug 26;20(8):e1012488. doi: 10.1371/journal.ppat.1012488 (PMC11379400; doi:10.1371/journal.ppat.1012488)

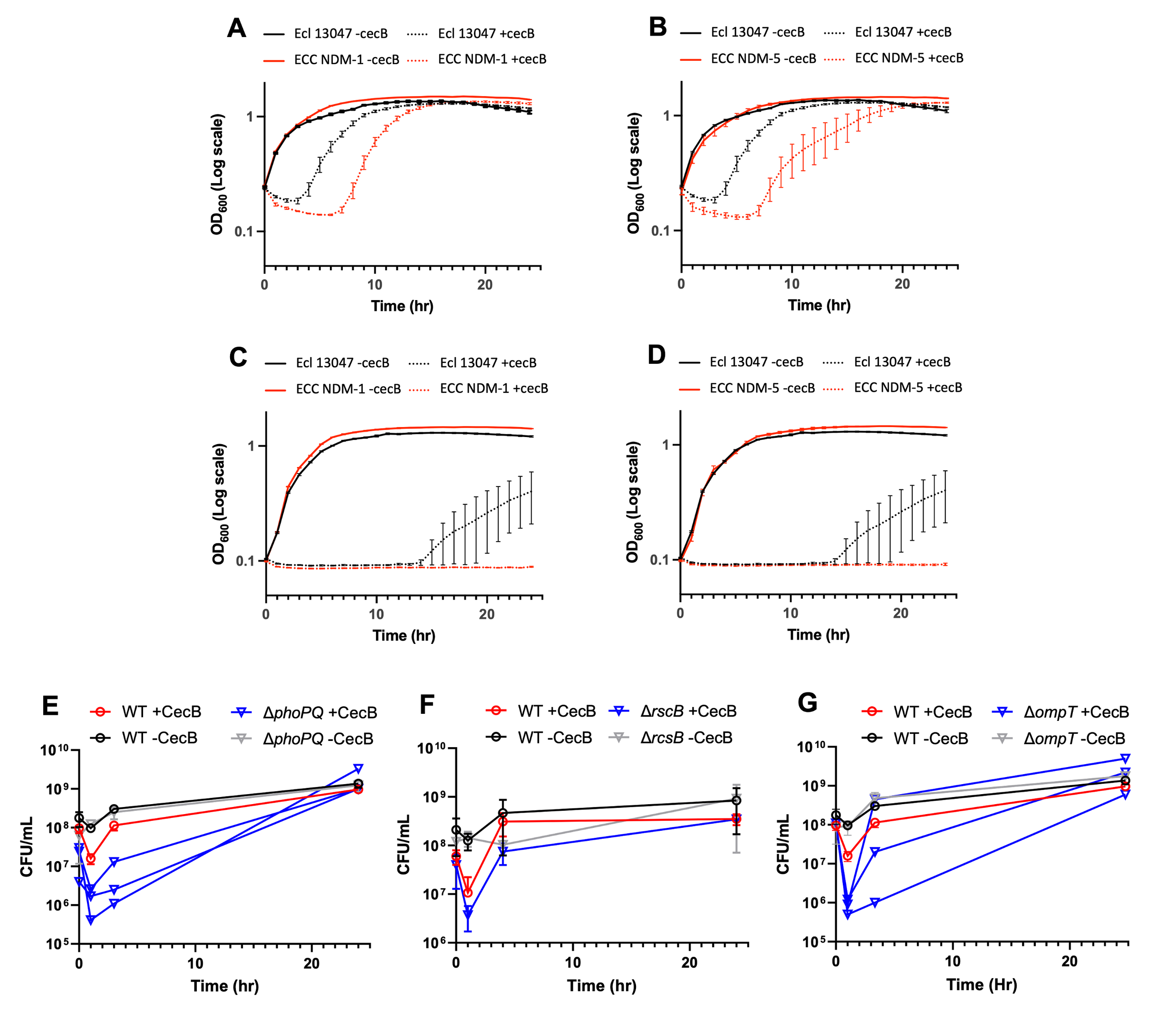

Supplement: S1 Fig — Overnight cultures of MDR ECC encoding NDM-1 or NDM-5 were diluted 10-fold (A and B) or 100-fold (C and D) into fresh LB containing 20 μg/mL cecB, and cells were grown at 37°C for 24 hours. OD600 measurements were taken every 10 minutes. Error bars represent standard deviation (n = 6). Overnight cultures of WT and (E) ΔphoPQ (F) ΔrcsB or (G) ΔompT were diluted 10-fold into fresh LB containing 20 μg/mL cecB, and cells were grown at 37°C for 24 hours. Samples were taken at select timepoints to quantify colony forming units (CFU) per mL. Error bars represent standard deviation (n = 3). In (C), individual replicates of ΔompT +cecB are shown due to high variance at the 3-hour timepoint. (TIF) [file ppat.1012488.s001.tif]

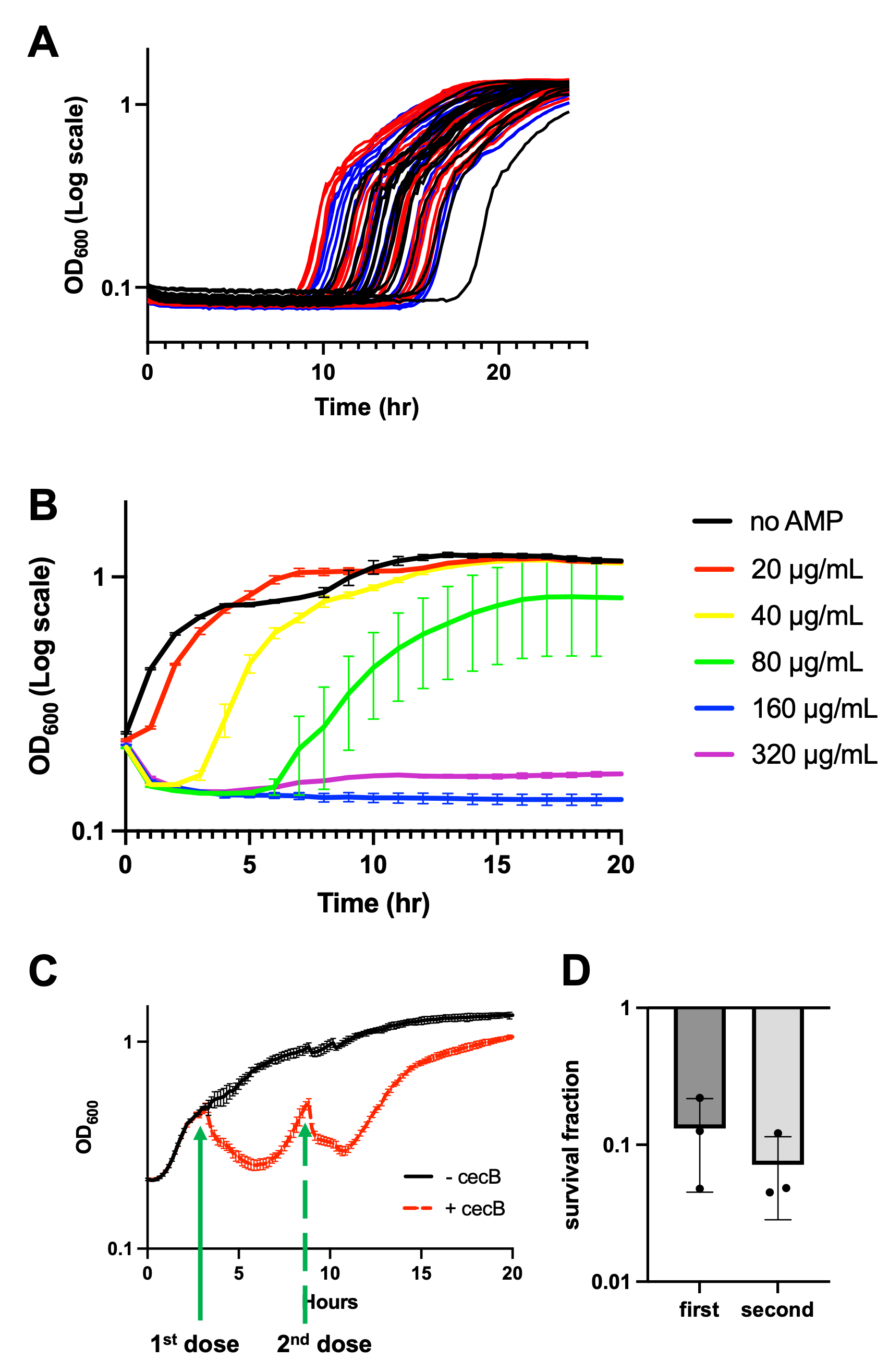

Supplement: S2 Fig — (A) WT cells from three independent wells in Fig 1A were sampled after 24 hours of growth in the presence of cecropin, grown overnight in LB, then re-exposed to 20 μg/ml cecB after 100fold dilution. Each of the 3 biological replicates is represented by a different color (black, red, blue), and each color contains 18 technical replicates. (B) Concentration-dependent growth and lysis. Overnight cultures were diluted 100fold into fresh medium containing the indicated concentration of cecB, growth (OD600) was measured in a plate reader. (C) Overnight cultures were diluted 100fold into fresh medium, grown to OD = 0.5, then cec B (2 x MIC) was added. Following regrowth to the same OD, cecB was again added at the same concentration (D) Cultures were treated as described in (C), but plated for CFU/mL after 1 hour of exposure to cecB. (TIF) [file ppat.1012488.s002.tif]

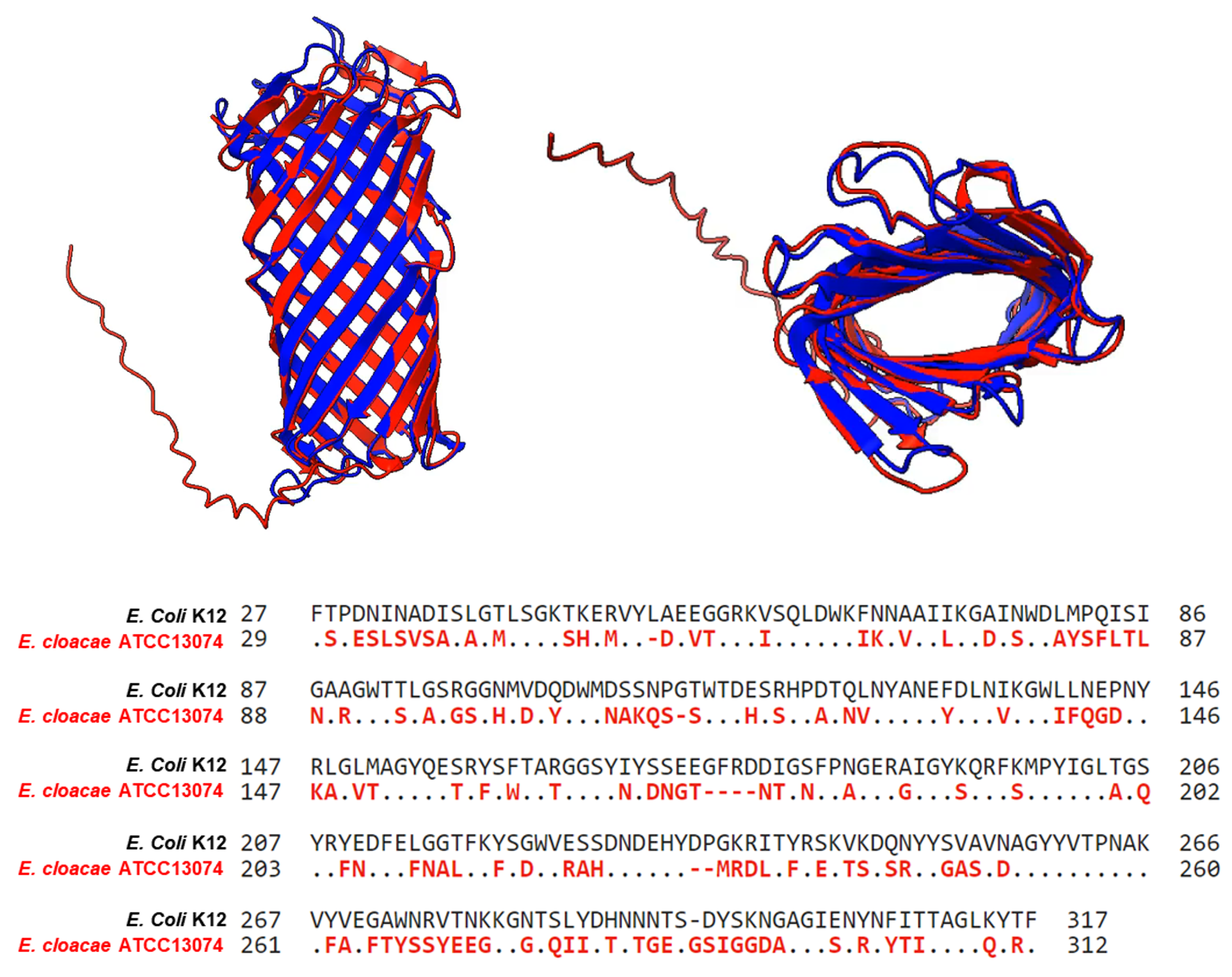

Supplement: S3 Fig — The crystal structure of E. coli OmpT (blue), overlaid with an AlphaFold prediction of the homologous OmpT we identified in E. cloacae (red). Pairwise amino acid sequence alignment revealed 50% identity. (TIF) [file ppat.1012488.s003.tif]

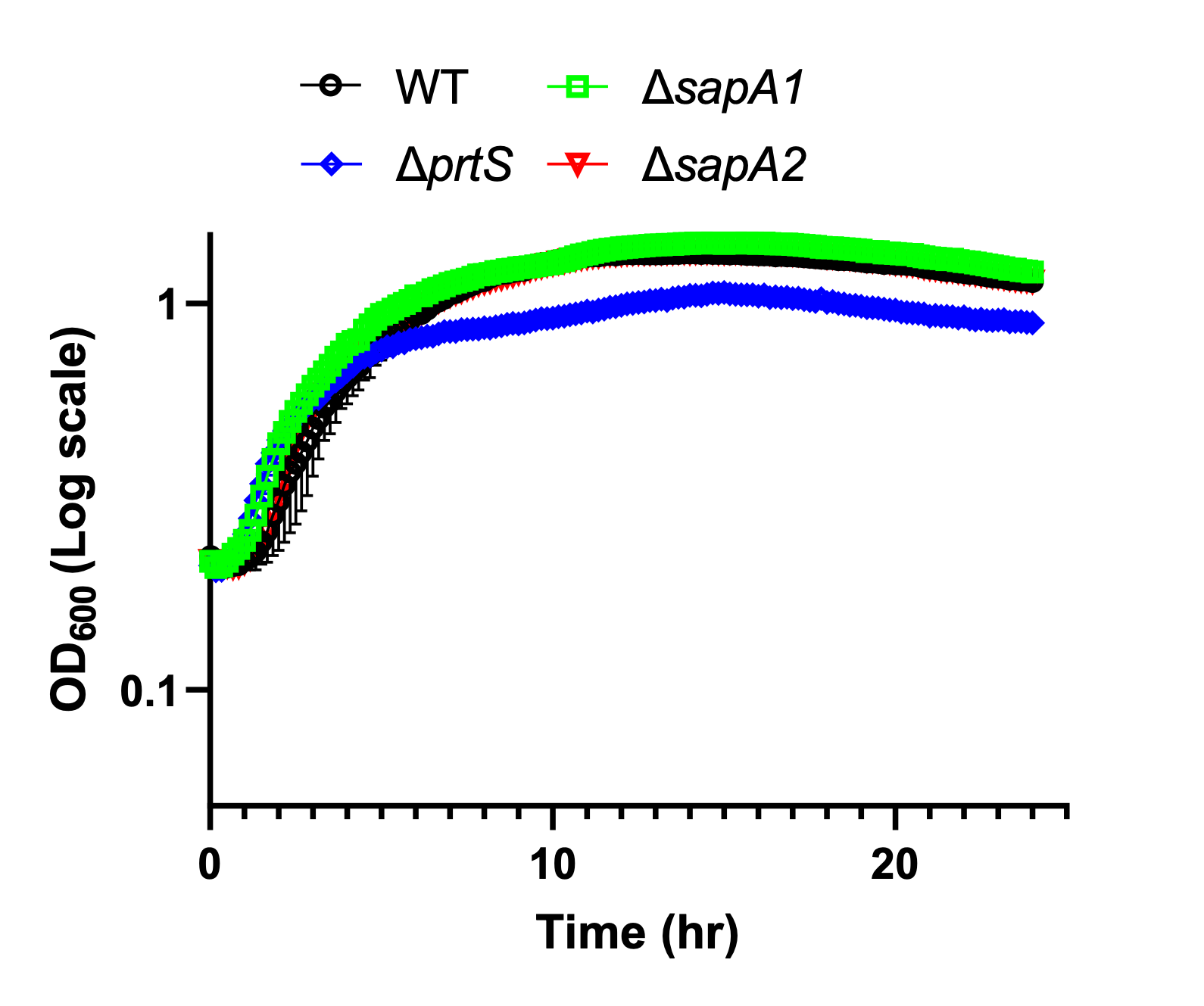

Supplement: S4 Fig — Overnight cultures were diluted 10-fold into fresh LB containing 20 μg/mL cecB, and cells were grown at 37°C for 24 hours. Error bars represent standard deviation (n = 3). (TIF) [file ppat.1012488.s004.tif]

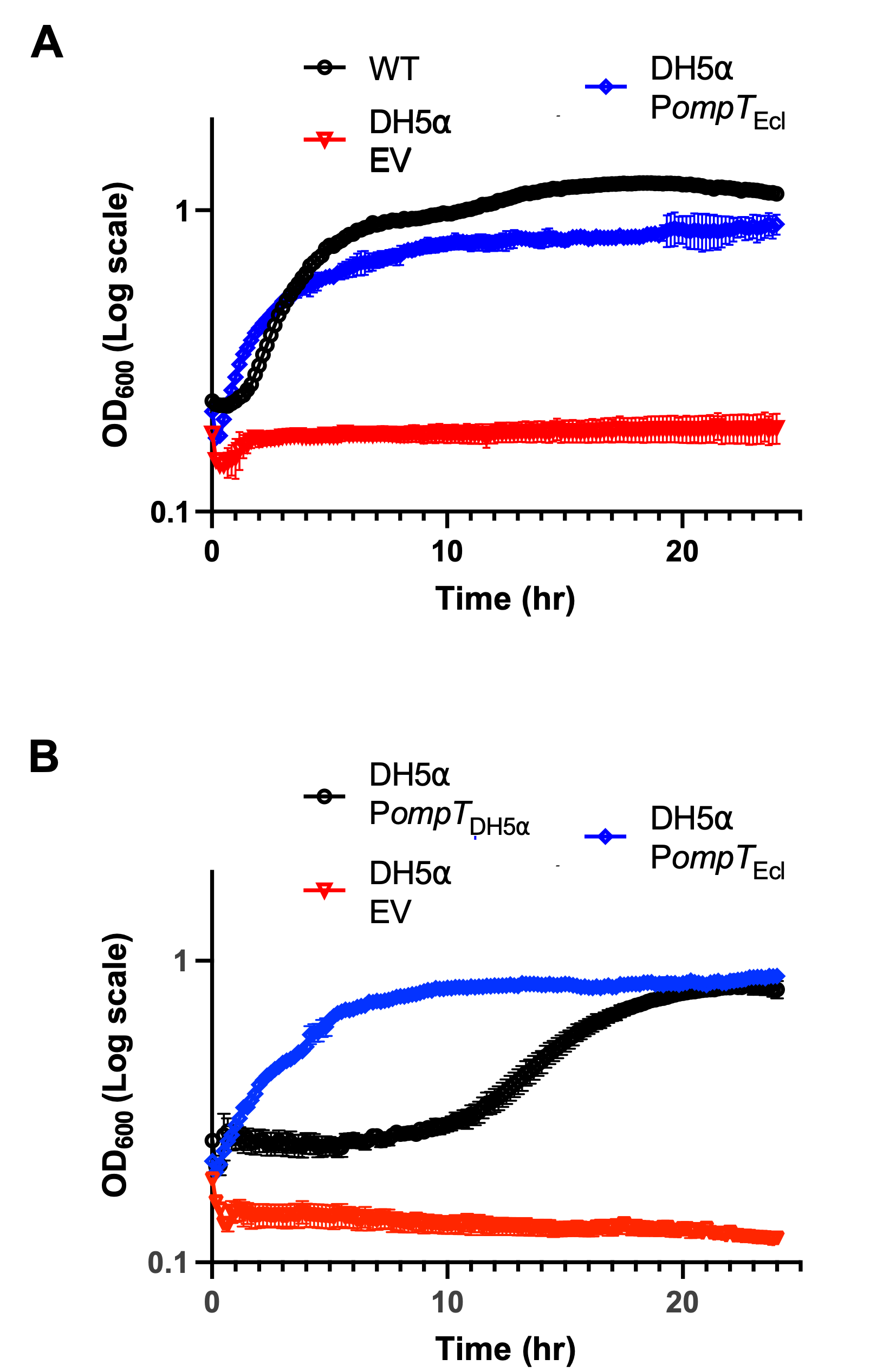

Supplement: S5 Fig — Overnight cultures were diluted 10-fold into fresh LB containing 20 μg/mL cecB, and cells were grown at 37°C for 24 hours. Error bars represent standard deviation (n = 3). (TIF) [file ppat.1012488.s005.tif]

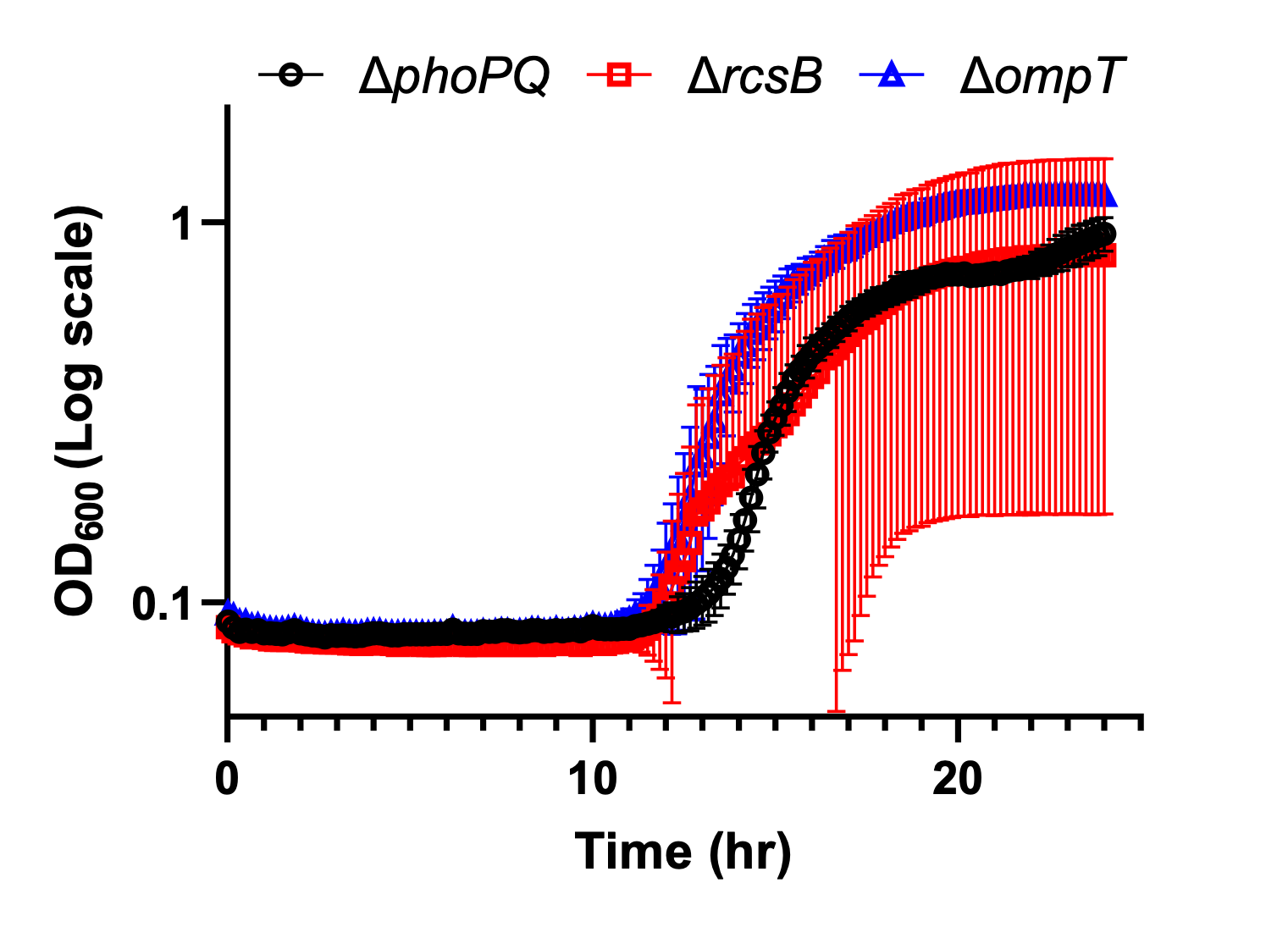

Supplement: S6 Fig — ΔphoPQ, ΔrcsB, and ΔompT were grown at 37°C in the presence of 20 μg/mL cecropin. After 24 hours, surviving cells were passaged ON in LB, then diluted 100-fold and re-exposed to 20 μg/mL cecropin. Cells were grown at 37°C for 24 more hours. Error bars represent standard deviation (n = 3). (TIF) [file ppat.1012488.s006.tif]

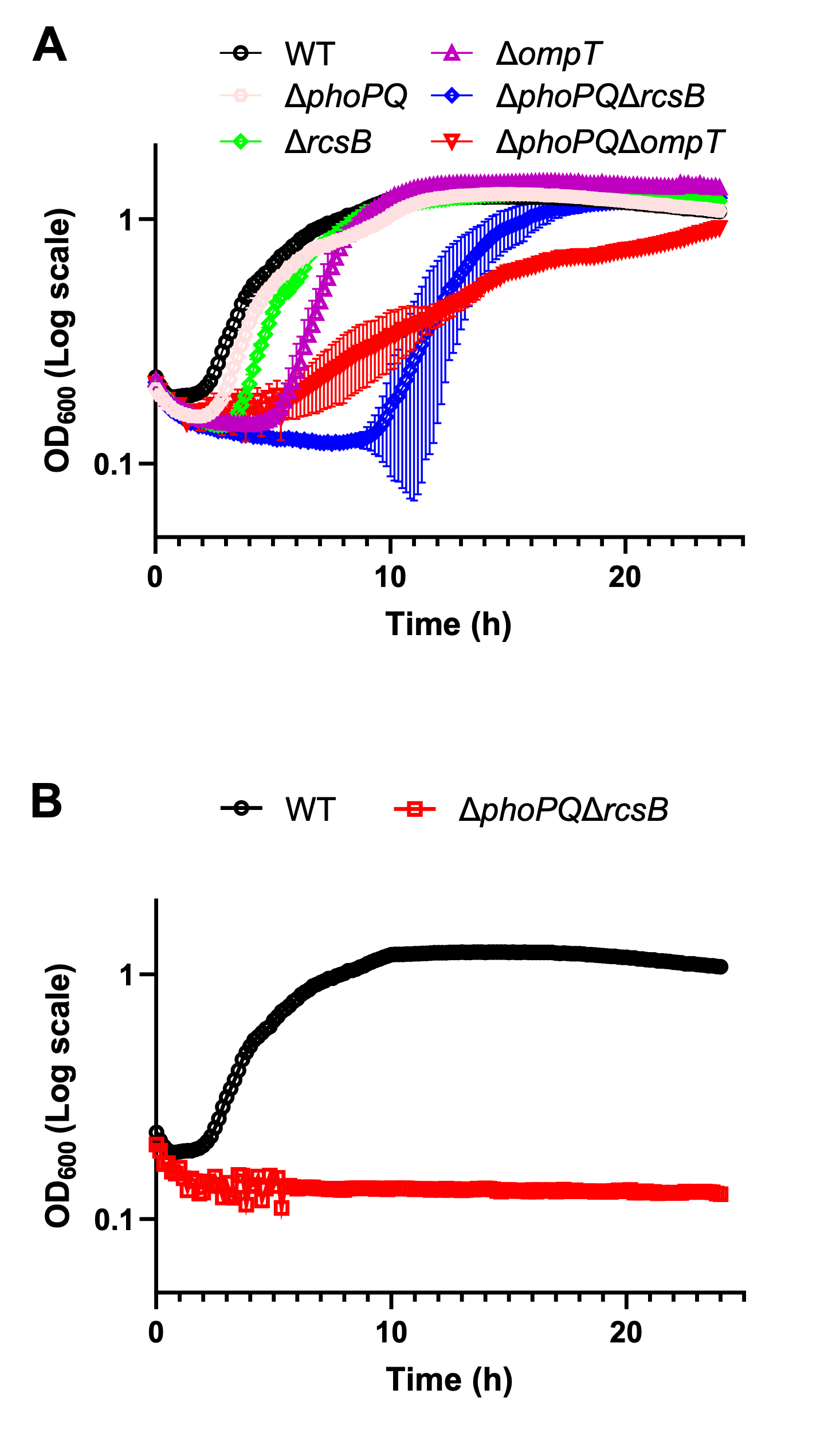

Supplement: S7 Fig — Overnight cultures were diluted 10-fold into fresh LB containing 20 μg/mL cecB, and cells were grown at 37°C for 24 hours. Error bars represent standard deviation (n = 3). (A) Representative growth curve of ΔphoPQΔrcsB and ΔphoPQΔompT response to cecB. (B) Example of replicate in which ΔphoPQΔrcsB mutant failed to grow in the presence of cecB. (TIF) [file ppat.1012488.s007.tif]

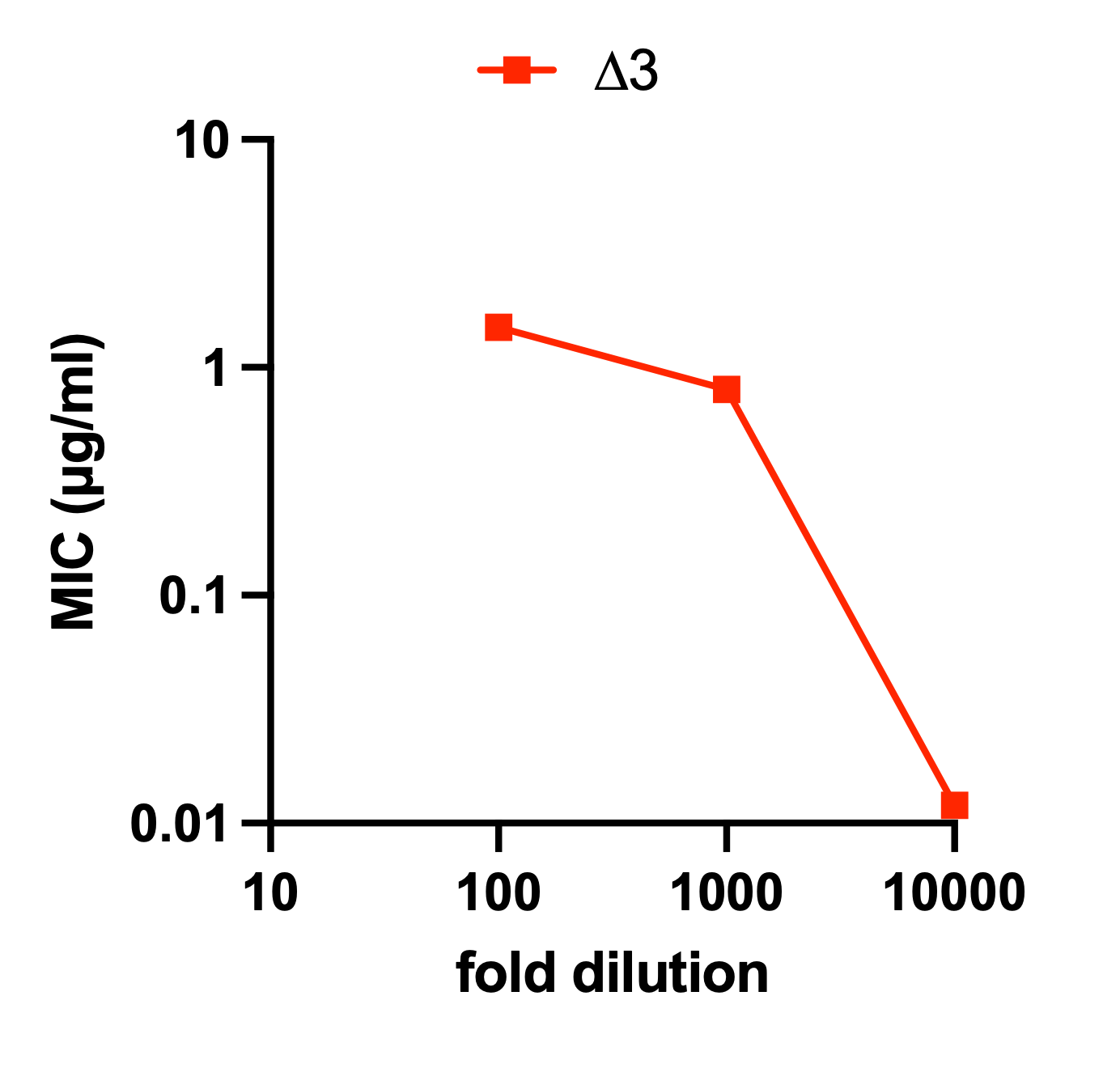

Supplement: S8 Fig — MIC assays were conducted at the indicated dilution of an overnight culture of E. cloacae Δ3 mutant. (TIF) [file ppat.1012488.s008.tif]
