# Supplementary material for: Multiple resistance factors collectively promote inoculum-dependent dynamic survival during antimicrobial peptide exposure in Enterobacter cloacae
Source: PLoS Pathog. 2024 Aug 26;20(8):e1012488. doi: 10.1371/journal.ppat.1012488 (PMC11379400; doi:10.1371/journal.ppat.1012488)
Supplement: S1 Table — (DOCX) [file ppat.1012488.s009.docx]

Table S1. Strains and plasmids used in this study

| **Strain or Plasmid** | **Antibiotic Resistance** | **Genotype or Plasmid Description** | **Reference** |
| --- | --- | --- | --- |
| **Strain** |  |  |  |
| *E. cloacae* strain ATCC 13047 |  | Wild type | [1] |
| *E. coli* strain DH5α |  | DH5α |  |
| ATCC 13047 Δ*phoPQ_Ecl_* |  | Δ*phoPQ_Ecl_* | [2] |
| ATCC 13047 Δ*phoPQ_Ecl_ /* pMMBKn::PhoPQ_Ecl_ | kan | Δ*phoPQ_Ecl_ /* pMMBKn::PhoPQ_Ecl_ | [2] |
| ATCC 13047 Δ*arnT_Ecl_* |  | Δ*arnT_Ecl_* | [2] |
| ATCC 13047 Δ*pagP_Ecl_* |  | Δ*pagP_Ecl_* | [3] |
| ATCC 13047 Δ*arnT_Ecl_* Δ*pagP_Ecl_* |  | Δ*arnT_Ecl_* Δ*pagP_Ecl_* | [3] |
| ATCC 13047 Δ*rcsB_Ecl_* |  | Δ*rcsB_Ecl_* | This study |
| ATCC 13047 Δ*ompT_Ecl_* |  | Δ*ompT_Ecl_* | This study |
| ATCC 13047 Δ*ompT_Ecl_ / pBAD::ompT_Ecl_* | chl | Δ*ompT_Ecl_ / pBAD::ompT_Ecl_* | This study |
| ATCC 13047 Δ*sapA1_Ecl_* |  | Δ*sapA1_Ecl_* | This study |
| ATCC 13047 Δ*sapA2_Ecl_* |  | Δ*sapA2_Ecl_* |  |
| ATCC 13047 Δ*prtS_Ecl_* |  | Δ*prtS_Ecl_* | This study |
| ATCC 13047 Δ*ompT_Ecl_ / pBAD::ompT_Ecl D104A,D106A_* | chl | Δ*ompT_Ecl_ / pBAD::ompT_Ecl D104A,D106A_* | This study |
| ATCC 13047 Δ*ompT_Ecl_ / pBAD::ompT_Ecl D226A,D228A_* | chl | Δ*ompT_Ecl_ / pBAD::ompT_Ecl D226A,D228A_* | This study |
| DH5α / *pBAD::ompT_Ecl_* |  | DH5α / *pBAD::ompT_Ecl_* | This study |
| ATCC 13047 Δ*phoPQ_Ecl_* Δ*rcsB_Ecl_* |  | Δ*phoPQ_Ecl_* Δ*rcsB_Ecl_* | This study |
| ATCC 13047 Δ*phoPQ_Ecl_* Δ*ompT_Ecl_* |  | Δ*phoPQ_Ecl_* Δ*ompT_Ecl_* | This study |
| ATCC 13047 Δ*phoPQ_Ecl_* Δ*rcsB_Ecl_* Δ*ompT_Ecl_* |  | Δ*phoPQ_Ecl_* Δ*rcsB_Ecl_* Δ*ompT_Ecl_* | This study |
| **Plasmids** |  |  |  |
| pMMB67EHKn:: | kan |  | [4] |
| pBAD33 | chl |  | [5] |

1. Guérin, F., et al., *Cluster-dependent colistin hetero-resistance in Enterobacter cloacae complex.* J Antimicrob Chemother, 2016. **71**(11): p. 3058-3061.

2. Kang, K.N., et al., *Colistin heteroresistance in Enterobacter cloacae is regulated by PhoPQ-dependent 4-amino-4-deoxy-l-arabinose addition to lipid A.* Mol Microbiol, 2019. **111**(6): p. 1604-1616.

3. Murtha, A.N., et al., *High-level carbapenem tolerance requires antibiotic-induced outer membrane modifications.* PLoS Pathog, 2022. **18**(2): p. e1010307.

4. Boll, J.M., et al., *A penicillin-binding protein inhibits selection of colistin-resistant, lipooligosaccharide-deficient Acinetobacter baumannii.* Proc Natl Acad Sci U S A, 2016. **113**(41): p. E6228-e6237.

5. Guzman, L.M., et al., *Tight regulation, modulation, and high-level expression by vectors containing the arabinose PBAD promoter.* J Bacteriol, 1995. **177**(14): p. 4121-30.
