# Supplementary material for: Multiple resistance factors collectively promote inoculum-dependent dynamic survival during antimicrobial peptide exposure in Enterobacter cloacae
Source: PLoS Pathog. 2024 Aug 26;20(8):e1012488. doi: 10.1371/journal.ppat.1012488 (PMC11379400; doi:10.1371/journal.ppat.1012488)
Supplement: S2 Table — (DOCX) [file ppat.1012488.s010.docx]

Table S2. Oligonucleotides used in this study

| ANM-4 | pMMBKn_seq_F | | cggttctggcaaatattctgaaa | |
| --- | --- | --- | --- | --- |
| ANM-5 | pMMBKn_seq_R | | gccgccaggcaaattc | |
| ANM-14 | sapA1-ver-F | | aaagcggcctacagtacgct | |
| ANM-15 | sapA1-ver-R | | ttgccgcaaacatcaggtctat | |
| ANM-16 | sapA1-F-1 | | ggcggggttttttcgttgatcacgtacgatacggtgtatgaaggctacgtc | |
| ANM-17 | sapA1-R-2 | | ttaaaagacaggaataatttttcactgcccgtcgtc | |
| ANM-18 | sapA1-F-3 | | gggcagtgaaaaattattcctgtcttttaattcgacggct | |
| ANM-19 | sapA1-R-4 | | cctgtacaccatgtgcaccggttcgaagatcctcaatctggtactccaccttag | |
| ANM-20 | sapA2-ver-F | | caattccgtgtaccgatgattatgt | |
| ANM-21 | sapA2-ver-R | | aagatggtttcggtcaggatcg | |
| ANM-22 | sapA2-F-1 | | ggcggggttttttcgttgatcacgtacgatgagaaccctgacaaagccaaaat | |
| ANM-23 | sapA2-R-2 | | gaacaccctactgaccctgacttcttcaaggaaatactc | |
| ANM-24 | sapA2-F-3 | | cttgaagaagtcagggtcagtagggtgttctccctct | |
| ANM-26 | ompT-ver-F | | ccccctttaccgccgttta | |
| ANM-27 | ompT-ver-R | | ccatttttttatacctttccggca | |
| ANM-28 | ompT-F-1 | | ggcggggttttttcgttgatcacgtacgatgcagaagatggctggaatagc | |
| ANM-29 | ompT-R-2 | | taaccacaacgggatttaatttccctaaatcaaatttaatcaatgcc | |
| ANM-30 | ompT-F-3 | | atttagggaaattaaatcccgttgtggttaatcaagat | |
| ANM-31 | ompT-R-4 | | cctgtacaccatgtgcaccggttcgaagattattgtttgcgcgctctgaatct | |
| ANM-40 | new-prtS-ver-F | | gaataagccaccaggagaaa | |
| ANM-41 | new-prtS-ver-R | | tttgttatcggtattctctgtg | |
| ANM-42 | new-prtS-F-1 | | atgaagatctgggataaacgatagac | |
| ANM-43 | new-prtS-R-2 | | gcaactctcctgttttctgg | |
| ANM-44 | new-prtS-F-3 | | atgcaggttccggaactga | |
| ANM-45 | new-prtS-R-4 | | attttgatgctgacgctgg | |
| ANM-78 | rcsB-del-1-F | | ggcggggttttttcgttgatcacgtacgatgtcgaattctgctgatgattattc | |
| ANM-79 | rcsB-del-2-R | | cacccgttctggagtattaggctaccttgctacagc | |
| ANM-80 | rcsB-del-3-F | | gcaaggtagcctaatactccagaacgggtgtgc | |
| ANM-81 | rcsB-del-4-R | | cctgtacaccatgtgcaccggttcgaagatacccgttcttccaggtaggta | |
| ANM-130 | wcaJ-flank-F | | caccagcacaaaaacaaatatcc | |
| ANM-131 | wcaJ-flank-R | | gtggagagatcaacagcaaactg | |
| ANM-126 | wcaJ-down-1-F | | ttgtaggccaggttataacccc | |
| ANM-127 | wcaJ-down-2-R | | gatgagcttacgtgaaaaaacca | |
| ANM-128 | wcaJ-up-3-F | | agagattaacgatgcattcgttct | |
| ANM-129 | wcaJ-up-4-R | | gtggagagatcaacagcaaactg | |
| ANM-146 | ompT-pBAD-D104A-D106A-F | | cgcCTGGATGAATGCAAAACAATC | |
| ANM-147 | ompT-pBAD-D104A-D106A-R | | taggCATCCATATGACCCGAGC | |
| ANM-148 | ompT-pBAD-D226A-H228A-F | | ggcCTATATGCGCGATCTGACCTTCC | |
| ANM-149 | ompT-pBAD-D226A-H228A-F | | tcggCGTTGTCGTGCGCGCGTA | |
|  | |  | |  |
|  | |  | |  |
