# Supplementary material for: Multiple resistance factors collectively promote inoculum-dependent dynamic survival during antimicrobial peptide exposure in Enterobacter cloacae
Source: PLoS Pathog. 2024 Aug 26;20(8):e1012488. doi: 10.1371/journal.ppat.1012488 (PMC11379400; doi:10.1371/journal.ppat.1012488)
Supplement: Attachment — Submitted filename: Murtha_reviewerRebuttal.docx [file ppat.1012488.s013.docx]

Reviewer Comments:

Reviewer's Responses to Questions

**Part I - Summary**
Please use this section to discuss strengths/weaknesses of study, novelty/significance, general execution and scholarship.

Reviewer #1: The manuscript by Murtha et al describes how Enterobacter populations resist killing by the antimicrobial peptide cecB. This study is important for numerous reasons. Antimicrobial peptides are a critical component of the innate immune response and resistance to or evasion of AMPs is necessary for to establish infection. Furthermore, AMPs are being explored as new treatment options, with some currently in clinical trials for the treatment of Gram-negative infection. Here, the authors characterize the response of Enterobacter to cecB and observe initial killing followed by delayed regrowth of the population. Interestingly, this phenomenon and the length of the lag phase following cecB treatment was heavily dependent on populations density and exhibited the hallmarks of heteroresistance. The authors then explored 3 hypotheses to uncover the mechanism of resistance, mutating genes responsible for three different established mechanisms of AMP resistance described in other organisms. Intriguingly, all three systems contributed to survival and regrowth of the population, likely due to heterogenous expression levels of each of the genes resulting in a sub-population exhibiting increased resistance to the AMP. Overall, this is a very well written, thorough and elegant paper that serves as a very useful addition to our knowledge of AMP resistance mechansims in an important pathogen.

Reviewer #2: The manuscript focuses on heteroresistance of E. cloacae to a cationic antimicrobial peptide (AMP) cecropin B. The authors demonstrate heteroresistance with growth curve experiments that show no heritable resistance occurs after challenge with cecropin B. This effect is inoculum dependent. The authors also confirm their data with CFU counts and quantitative microscopy. Then, the authors test 3 known systems in other bacteria that antagonize AMPs, PhoPQ, RcsB, and OmpT, in E. cloacae. The knockout of each of these systems results in reduced heteroresistance, allowing the authors to conclude that heteroresistance to cecropin B in E. cloacae is multifaceted. The work is done carefully with good controls, but I have two concerns about the impact/importance of the results.

First, all experiments were done on the type strain of E. cloacae rather than MDR strains with more clinical/health relevance.

Response: We chose the type strain since this study focuses on proof of principle. We chose to map out AMP resistance mechanisms in an opportunistic pathogen, and the well-characterized type strain struck us as an ideal starting point. We have now also included two MDR clinical isolates of *E. cloacae* (see new Fig. S1 A-D) where we see the same dynamic survival as in ATCC 13047.

Second, the authors do not motivate their choice of cecropin B sufficiently. Of all of the AMPs out there, why choose this one? It does not appear to be particularly potent, and it is a linear peptide which will impact its stability (indeed, likely observed in the authors’ OmpT experiments).

We chose cecropin because it a) consistently kills bacteria in a variety of growth media (unlike many other AMPs that often require specialized conditions for their lethal activity, making it uniquely suited for establishing fundamental mechanisms, b) recapitulates the mechanism of action of many other LPS-targeting AMPs, and c) it has special clinical relevance since cecropins are under active development as clinical therapeutics. We have added a more thorough discussion motivating our choice of AMP to the introduction in the revised manuscript.

Besides these overarching questions, there are several further questions/concerns that should be addressed in a revised paper.
1) Introduction: this section should include information about the sequence of cecropin B and its helical structure as well as if anything is known about whether it forms pores in membranes or kills by other mechanisms

Done!

2) Line 54: I am intrigued by the statement that AMPs kill faster than antibiotics. Could the authors discuss this more and back it up with numbers?

This refers to a common property of AMPs to accumulate on membranes and then cause instantaneous death once a lethal threshold is reached. Compare this, for example, with beta lactam antibiotics, where cells undergo significant cell envelope expansion before lysing (if they lyse at all). We have cited relevant papers on this in the manuscript.

3) Line 86: while the intro is already on the long side, I would like the authors to add a few more sentence talking about the specifics of how CPS is regulated by Rcs. Up or down-regulated? Which polysaccharides?

We have added this information to the introduction.

4) Line 109: state the values of the “low MICs”

We deleted this statement, since upon further consideration, we realize that “low” is not an easily-definable quantity.

5) Line 114: report the concentration of cecropin B used

Done!

6) Line 128: I was confused about referring to the 1000-fold dilution as a standard condition because the paragraph only discusses the 10 and 100-fold dilutions

Yes, this is a bit of an arbitrary choice. We could as well have chosen the higher dilutions as the “standard”, but argue that at lower dilution, there are fewer confounding variables (inoculum effect, protease effects).

7) Line 184-185: amino arabinose is not an enzyme. Please correct and also state briefly how this enzyme leads to AMP resistance

Corrected and expanded!

8) Line 212: can you define the various components of the capsule, especially if there are aspects of it that are specific to E. cloacae?

We have added some details on the type of capsule that the Rcs system controls.

9) Line 260: did the authors consider overexpression of the DH5a OmpT in E. cloacae to rule out the possibility that the lack of cleavage in DH5a is just due to lower expression?

We have done this experiment (see new Fig. S5B) and found that even upon overexpression, *E. coli*’s native OmpT does not confer cecropin resistance to the same degree as *E. cloacae* OmpT.

10) Line 269: it should be possible to use LC-MS to try to quantify the degradation of cecropin B; was this considered?

We have indeed attempted this, but hit several roadblocks in trying to quantify the peptide from supernatants and lysates, leaving the results largely uninterpretable. Note, however, that the presence or absence of cecropin at the end of the experiment does not affect any of our conclusions.

Reviewer #3: Review: Murtha et al.

Overall, the experiments we well described, and the data clearly reported. The novelty is diminished by the target gene approach which only looked at known mechanisms, but it was still fruitful. The generalizability was diminished by the focus on just on AMP, and one bacterial strain. I believe there are a there are a few points of clarification, mostly on the interpretation that could make the paper stronger.

We thank the reviewer for their constructive comments!

1. The justification of investigating this specific AMP is not made. Given the large number of AMPs that could have been chosen. It would have made a more compelling paper I the authors better explained why this AMP is of particular interest.

Please see response to reviewer 2. We have now included a stronger justification for why we believe cecropin B is an ideal model peptide in the revised manuscript.

2. Their interpretation of the population level of heteroresistance assays in the results section is problematic. Specifically, the delayed growth of the population after initial decline. The authors do not address until much later, in the discussion, the clear alternative that the level of cecropin in the media is being depleted in a manner dependent upon the E. cloacae concentration. If this is the case, one could imagine there is no phenotypic heterogeneity among the E. cloacae within the culture, but simply a stochastic killing of cells until the cecropin level drops to a point where the cell can grow again.

-- Similarly, in the single cell microscopic studies, I don’t see that they fully explain why their findings may not be due to stochastic effects, and potentially local heterogeneity in cecropin concentration in the LB pads, which presumable could develop rather quickly if the E. cloacae are inactivating the cercopin.
-- The authors then lean heavily into the model into which cecropin is being depleted in the discussion ( eg line 348. ), which is also consistent with their data, but confusing given the inconsistency with how the data are interpreted in the results section.

Yes, we do believe that the cecropin either gets sequestered or degraded. However, this would still indicate population heterogeneity: Why does the minority population survive long enough to regrow after the cecropin is removed? More importantly, our single-cell analyses on agarose pads clearly indicate that this subpopulation is a dynamically growing and dying population, in which some individuals keep lysing even after regrowth. Thus, the survivors clearly deserve the “heterogeneity” label. Perhaps this is a semantic distinction: We use “heterogeneous” to refer to phenotypic outcomes, rather than to indicate underlying physiology (which we do not currently understand mechanistically). To address the confusion of our model appearing only in the discussion section, we have now added a sentence to the results section foreshadowing that cecropin likely disappears during the course of our experiment.


-- Overall, I felt the frequent use of the term heteroresistance detracted from the paper, and I see no compelling evidence that heteroresistance (if defined as a phenotypic state the is vertically passed down for at least a few cell division cycles) exists.

We agree with the reviewer and actually went back and forth a bit about using this term. In the end, we settled for “dynamic survival” when referring to single-cell behavior and simply “resistance” when referring to the population. In the revised manuscript, we streamlined the text to focus on this term, and now mention “heteroresistance” only as a point of comparison to this phenomenon.

3. The genetic studies including identification of the ompTecl with exploration to mutation in the active site was strong and well done.

Thank you!

**Part II – Major Issues: Key Experiments Required for Acceptance**
Please use this section to detail the key new experiments or modifications of existing experiments that should be absolutely required to validate study conclusions.

Generally, there should be no more than 3 such required experiments or major modifications for a "Major Revision" recommendation. If more than 3 experiments are necessary to validate the study conclusions, then you are encouraged to recommend "Reject".

Reviewer #1: I have no major criticisms.

Reviewer #2: see above

Reviewer #3: None

**Part III – Minor Issues: Editorial and Data Presentation Modifications**
Please use this section for editorial suggestions as well as relatively minor modifications of existing data that would enhance clarity.

Reviewer #1: Can the authors more specifically introduce what is known about the mechanism by which cecropins kill. In the discussion, it may be worthwhile suggesting that the resistance mechanisms uncovered here may play more or less of a role in resistance to other AMPs depending on their mechanism of action.

Done!

The impact of population density on the outcome of AMP treatment is striking. Perhaps the authors would care to comment on the implications of this finding for the establishment of infection or for the use of AMPs to as new anti-infectives?

Done!

In Figure 7, 2. Membrane Stress/AMPs, consider removing AMPs as that’s already in the figure.

We think this is important to keep, to indicate that PhoPQ can sense the presence of AMPs directly, or indirectly through general cell envelope stress.

Reviewer #2: see above

Reviewer #3: none
